# Inhibition of Both EGFR and IGF1R Sensitized Prostate Cancer Cells to Radiation by Synergistic Suppression of DNA Homologous Recombination Repair

**DOI:** 10.1371/journal.pone.0068784

**Published:** 2013-08-12

**Authors:** Yong Wang, Jian Lin Yuan, Yun Tao Zhang, Jian Jun Ma, Peng Xu, Chang Hong Shi, Wei Zhang, Yu Mei Li, Qiang Fu, Guang Feng Zhu, Wei Xue, Yong Hua Lei, Jing Yu Gao, Juan Ying Wang, Chen Shao, Cheng Gang Yi, He Wang

**Affiliations:** 1 Department of Urology, Tangdu Hospital, Fourth Military Medical University, Xi'an, Shaan'xi Province, P.R. China; 2 Department of Urology, Xijing Hospital, Fourth Military Medical University, Xi'an, Shaan'xi Province, P.R. China; 3 Department of Medicine and Training, Tangdu Hospital, Fourth Military Medical University, Xi'an, Shaan'xi Province, P.R. China; 4 Department of Experimental Animals, Fourth Military Medical University, Xi'an, Shaan'xi Province, P.R. China; 5 Department of Plastic Surgury, Xijing Hospital, Fourth Military Medical University, Xi'an, Shaan'xi Province, P.R. China; Thomas Jefferson University, United States of America

## Abstract

Reduced sensitivity of prostate cancer (PC) cells to radiation therapy poses a significant challenge in the clinic. Activation of epidermal growth factor receptor (EGFR), type 1 insulin-like growth factor receptor (IGF1R), and crosstalk between these two signaling pathways have been implicated in the development of radiation resistance in PC. This study assessed the effects of targeting both receptors on the regulation of radio-sensitivity in PC cells. Specific inhibitors of EGFR and IGF1R, Erlotinib and AG1024, as well as siRNA targeting EGFR and IGF1R, were used to radio-sensitize PC cells. Our results showed that co-inhibiting both receptors significantly dampened cellular growth and DNA damage repair, and increased radio-sensitivity in PC cells. These effects were carried out through synergistic inhibition of homologous recombination-directed DNA repair (HRR), but not via inhibition of non-homologous end joining (NHEJ). Furthermore, the compromised HRR capacity was caused by reduced phosphorylation of insulin receptor substrate 1 (IRS1) and its subsequent interaction with Rad51. The synergistic effect of the EGFR and IGF1R inhibitors was also confirmed in nude mouse xenograft assay. This is the first study testing co-inhibiting EGFR and IGF1R signaling in the context of radio-sensitivity in PC and it may provide a promising adjuvant therapeutic approach to improve the outcome of PC patients to radiation treatment.

## Introduction

Prostate cancer (PC) is the most common malignancy and the second leading cause of cancer-related deaths among male patients [1]. During cancer progression, the initial growth of PC cells is androgen-dependent, and these cells undergo apoptosis upon androgen depletion. As a consequence, androgen ablation was considered the standard treatment for PC for over 50 years [2]. Many patients eventually developed a hormone-refractory disease due to the growth of androgen-refractory cancer cells, which leads to failure of androgen ablation therapy and leaves patients with fewer therapeutic options [3], [4]. Combination of definitive local therapies, such as radical prostatectomy together with adjuvant radiotherapy, has been demonstrated to improve the survival of PC patients [5], [6]. However, such therapy is challenged by the emergence of resistance in tumor cells. It is, therefore, of paramount importance to develop novel therapeutic strategies to overcome radioresistance and improve radio-sensitivity by targeting molecular machineries in androgen-independent PC cells.

Epidermal growth factor receptor (EGFR) and insulin-like growth factor receptor (IGF1R), two most important tyrosine kinase receptors, play critical roles in cancer development and progression through the regulation on cell proliferation, apoptosis, anchorage-independent growth, invasion, angiogenesis, cancer immunity and resistance to chemo- and/or radiotherapy [7]. These two receptors are frequently overexpressed in a variety of human cancers including PC [8], [9], [10], and therefore could be used as candidates for targeted cancer therapy. Indeed, inhibitors of EGFR and another EGFR family member Her2, including Erlotinib, Lapatinib, Cetuximab, and Gefitinib, are the most successful options in current clinical treatment of different human cancers, As expected however, the development of *de novo* resistance has been observed in clinic after long-term use of these medicines, suggesting the existence of bypass mechanisms within tumor cells [11]. Mechanistic studies on the cellular and molecular events revealed that extensive crosstalk between EGFR and IGF1R signaling occurs at multiple levels, and that blockage of EGFR signaling leads to enhanced responses to the IGF1R ligand, IGF [12], [13]. These data imply that targeting both receptors at the same time could provide better efficacy in cancer treatment and overcome tumor resistance to an individual inhibitor, while improving the sensitivity of individual inhibitors to cancer therapy. Consistently, studies have shown that dual targeting of both receptors blocks their reciprocal hyperphosphorylation, inhibits the proliferation and induces apoptosis in multiple cancer cells including PC and colorectal cancer [14], [15].

In this study, we assessed the effects of targeting both EGFR and IGF1R signaling in the responses of PC cells to γ-irradiation. Our data demonstrated the potency of targeting both pathways in modulating the behaviors of PC cells following radiotherapy and revealed the underlying mechanisms. This is a seminal study that further justifies the combinatorial use of inhibitors for EGFR and IGF1R pathways in the treatment of PC.

## Materials and Methods

### Cell culture and treatment

The human androgen-independent PC cells DU145, PC3, ARCaP_E_ and ARCaP_M_ and human normal prostate epithelium cell line PrEC were purchased from American Type Culture Collection (Manassas, VA, USA). The R503 was from the Experimental Animal Center of the Fourth Military Medical University. The cells were treated with dimethyl sulfoxide (DMSO, as the vehicle control), 10 µM Erlotinib (EGFR inhbibitor, Eton Bioscience, San Diego, CA) and/or 10 µM AG1024 (IGF1R inhibitor, Santa Cruz Biotechnologies, Santa Cruz, CA) (as experimental groups) for 1 h. Cells were irradiated as described by Liu et al [16]. In some experiments, the cells were also transfected with IRS1 or non-silencing control (NSC) siRNAs (50 nM, Invitrogen, Shanghai, China) according to manufacturer's protocol.

To establish irradiation-tolerant sublines, PC cells were irradiated at 2 Gy per day, 5 days a week in a FCC-8000C ^60^Co irradiator. After six months, the sublines of DU145 that survived ionized irradiation were established and named DU145-IIRR. These sublines were then subjected to drug treatment and further experiments.

### Clonogenic assay

The synergistic colony formation following the combined treatment with of EGFR and IGF1 inhibitors and irradiation was investigated by monolayer clonogenic assays. Cells were serum-starved overnight. Five thousand cells were seeded into 10-cm-diameter tissue culture dishes with 10-mL medium. The cells were treated with AG1024 or Erlotinib for 1 h and irradiated at indicated dosage after they had adhered to the dishes. Colony formation was determined with crystal violet staining by using a Coulter particle counter on day 10 after cell seeding. Surviving fraction was defined as the cloning efficiency of the treated cells divided by that of the control cells. Experiments were repeated three times.

### Flow cytometry assay

To analyze cell apoptosis, cells (1×10^4^/well) were plated onto 6-well plates. After 24 h, cells were serum starved for 24 h, and then treated with either AG1024 or Erlotinib for 1 h. Cells were then irradiated at the dosage of 2 Gy. At 4 h after irradiation, the cells were harvested by trypsinization, fixed in 70% ethanol at 4°C for 2 h and washed in 5 mL of PBS. Cells were then stained with 1 mL of propidium iodide (PI) solution (0.2 mg of RNAse A, 0.02 mg PI, and 1 mL Triton X-100). The DNA content in different cell-cycle phases was determined by FACS flow cytometer (BD Biosciences, San Jose, CA). To detect apoptotic rate in treated cells, the cells were stained with Annexin V and PI, and then subjected to flow cytometry as described [16]. The Annexin V-positive and PI-negative cells undergoing early apoptosis were counted as apoptotic cells. All experiments were conducted in duplicates and data shown are representative of three independent experiments.

### Protein extraction and Western blotting

Different groups of cells were lysed with 1% SDS lysis buffer (Beyotime Inc., Beijing, China). For nuclear protein extraction, the cells were washed with hypotonic lysis buffer (Teknova, Beijing, China), and dounced in the cell douncer (Wheaton Ltd, Millville, NJ). The nuclear components were then separated from cytosolic extracts by centrifugation, followed by lysis in RIPA buffer (Pierce Inc., Beijing, China).

Immunoprecipitation and Western-blot assay were performed as described by Liu et al [17]. The following antibodies were from Cell Signaling: anti-γH2AX, anti-IRS1, anti-phospho IRS1(pY612), anti-IGF1R, anti-phospho IGF1R, anti-EGFR, anti-phospho EGFR(Y1068), anti-β-tubulin, anti-ERK, anti-phospho-ERK, anti-AKT, anti-phospho AKT(S473), anti-DNA-PK, anti-Ku70, anti-Ku80, and anti-XRCC4. The anti-Lamin antibody was from Abnova (Shanghai, China). The anti-HP-1α antibody was from Millipore (Shanghai, China). β-tubulin, Lamin and HP-1α were used as loading controls.

### Immunofluorescence

The γH2AX and Rad51 foci formation was investigated according to Barber et al [18]. For immunofluorescence staining for γH2AX and Rad51, cells were fixed in 1% paraformaldehyde for 10 min at room temperature followed by 70% ethanol for 10 min at room temperature. After wash with PBS containing 0.1% Triton for 10 min, cells were permeabilized with 0.5% Triton in PBS for 10 min at room temperature. After three washes in PBS, cells were blocked with 5% bovine serum albumin (BSA) in PBS for 60 min. Then anti-γH2AX (Trevigen, Gaithersburg, MD, 1∶2000) or anti-Rad51 antibody (Oncogene research, 1∶300) was added in 5% BSA in PBS and incubated with cells at 4°C overnight with gentle shaking. After four washes in PBS, cells were incubated in the dark with a FITC-labeled secondary antibody in 5% BSA at a dilution of 1∶2000 for γH2AX and 1∶200 for anti-Rad51 detection for 1 h at room temperature. Following four more washes in PBS, the nuclei were stained in the dark with 4′,6-diamidino-2-phenylindole (DAPI) (1 µg/mL, Invitrogen) in PBS for 5 min, and coverslips were mounted with Fluoromount G (Southern Biotech., Birmingham, AL). Slides were examined on a Leica fluorescence microscope, with images captured by a CCD camera and imported into the Advanced SPOT Image analysis software (SPOT Imaging Solutions, Sterling Heights, MI). For each treatment condition, the γH2AX or Rad51 signals were determined in at least 50 cells. All observations were validated from at least three independent experiments.

### Assays for HR-directed DNA repair (HRR) and the non-homologous end joining (NHEJ)

The quantitative *in vitro* homologous recombination (HR) assay was performed using the pDR-GFP recombination reporter system [19]. Briefly, the pDR-GFP plasmid expressing two nonfunctional GFP genes was stably transfected into DU145 cells. A second plasmid encoding the restriction enzyme I Sce-I and a third plasmid expressing red fluorescent protein (RFP) with a mitochondrial localization signal to indicate transfection efficiency were transiently transfected into DU145 cells containing the pDR-GFP plasmid. When I-SceI was expressed, it produced DNA double-strand breaks (DSBs) within the SceGFP fragment and stimulated HRR to restore intact GFP gene. DNA repair by HRR was evaluated by counting cells with both nuclear GFP signal and mitochondrial RFP signal vs. all positively transfected cells, that is, ratio of cells with both red and green signals to those with only red signals.

The cell-free NHEJ assay was performed as described previously [20] with nuclear extracts from 1×10^7^ DU145 cells.

### *In vivo* tumor radiation therapy

The animal experiments were approved by the Ethnic Committee of the Fourth Military Medical University (Xi'an, China). 5×10^6^ DU145 cells were pre-mixed with Matrigel (BD Biosciences) for subcutaneous injection into the flank of 10-week-old female nude mice. Twenty-two days later (Day 0), the mice were randomly divided into 5 groups, 5 mice per group, based on the treatments they would receive: i.e., control group received no treatments; IR group received γ-irradiation of different dosages on days 3, 6, 8, and 10, respectively; IR+Erlotinib group received γ-irradiation as the irradiation group, plus 100 mg/kg/d of oral Erlotinib for 10 days; IR+AG1024 group received γ-irradiation, plus100 mg/kg/d of oral AG1024 for 10 days; IR+AG1024 and Erlotinib received γ-irradiation, plus 100 mg/kg/d both drugs for 10 days. The tumor volume (V) was monitored every three days for seven weeks by measuring the length (L) and width (W), and calculated as V = 0.5×L×W^2^. The result was expressed as proliferation index (PI, PI = V treatment/V control).

### Statistical analysis

The effect of Erlotinib and AG1024 on inhibition of cell proliferation, xenograft growth, clonogenic survival, and caspase activation was analyzed statistically using an unpaired two-tailed Student's *t* test. All quantitative data were presented as mean ± SD from at least three independent experiments for *in vitro* experiments or from all animals within the group for *in vivo* experiments. P value of ≤0.05 was considered statistically significant. * and ** was labeled for P<0.05 and P<0.01 respectively, as compared to the control cells in all the figures.

## Results

### Reduction of tumor cell viability and induction of apoptosis by targeting of EGFR and/or IGF1R before irradiation treatment

**Figure 1:**
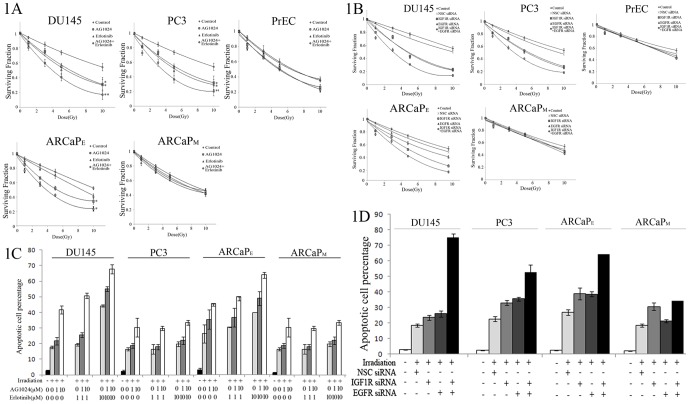
Effects of EGFR and IGF1R inhibitors in suppression of epithelial cancer cell through synergistic induction of DSB-related apoptosis. **A**, Radio-sensitivity assay of cells treated with EGFR and IGF1R inhibitors. Cancer cells of different groups were treated without (control) or with AG1024 (10 µM), Erlotinib (10 µM) or both for 1 h and then subjected to radio-sensitivity clonogenic assay. After 10 days, the clones were fixed and stained. Surviving fraction was calculated according to the colony counts and plating efficiency. **B**, Radio-sensitivity assay of cells treated with EGFR and IGF1R siRNAs. Cells of different groups were transfected with non-silencing control siRNA (NSC-siRNA), EGFR and/or IGF1R siRNA, and then subjected to radio-sensitivity clonogenic assay as described in panel A. **C**, Apoptosis of cells treated with EGFR and IGF1R inhibitors. Prostate cancer cell lines were pre-treated with AG1024 or Erlotinib at indicated concentrations for 1 h, and then irradiated for 2 Gy. After 4 hours, they were stained with Annexin V and propidium iodide for flow cytometry to determine percentage of apoptotic cells. **D**, Apoptosis of prostate cancer cell lines transfected with EGFR and/or IGF1R siRNA. Cells of different groups were transiently transfected with NSC siRNA, IGF1R siRNA or EGFR siRNA for 24 h, and then irradiated for 2 Gy. Apoptosis was analyzed as in panel C. The data were presented as mean ± SD from three independent experiments. *P<0.05, **P<0.01, as compared to the control cells.

In this study, we first assessed the induced sensitivity of different cancer cells to radiation *in vitro*. We grew and treated different cancer cell lines with Erlotinib (10 µM) and/or AG1024 (10 µM) for 1 h and then irradiated them with 2 Gy. Our data showed that tumor cell viability was significantly reduced and tumor cell apoptosis enhanced by irradiation following inhibition of both EGFR and IGF1R, as compared to irradiation treatment alone or irradiation plus blocking of either receptor (Figure 1A, B). Specifically, when compared to the vehicle-treated control cells, either AG1024 or Erlotinib significantly reduced tumor cell viability in epithelial PC cell lines (P<0.05), yet the most robust growth-inhibitory effect by irradiation was achieved with simultaneous application of both inhibitors (P<0.05, as compared to AG1024 or Erlotinib treatment in DU145, PC3 and ARCaP_E_ cells) (Figure 1A). In contrast, AG1024 or/and Erlotinib could not radio-sensitize normal prostate epithelium cell line PrEC (Figure 1A). Our data also showed that in both DU145 and PC3 cells, AG1024 significantly reduced the phosphorylation of IGF1R, whereas Erlotinib dramatically inhibited the phosphorylation of EGFR, and neither showing cross-activity on the other receptor, indicating that both inhibitors function potently and specifically (Figure S1).

Besides epithelial PC cells, we also assessed the effects of targeting both EGFR and IGF1R signaling in the radio-sensitization response of mesenchymal-like PC cells. For this purpose, we used ARCaP_M_, the mesenchymal counterpart of ARCaP_E_ developed by Graham et al [21]. Both cells lines exhibit minimal expression of androgen receptor [22]. Consistent with previous findings by Buck et al [14], combined treatment with EGFR and IGF1R inhibitors could not synergistically inhibit mesenchymal cell growth in response to irradiation, as it did for epithelial growth (Figure 1A).

To avoid the non-specific effects associated with small molecular inhibitors, we also repeated the experiment in Figure 1A using siRNA specific for EGFR and IGF1R (Figure 1B). As shown in Figure S2, both siRNA worked efficiently in knocking down IGF1R and EGFR, respectively. With single or dual knockdown of EGFR and/or IGF1R by siRNA, we obtained similar results as in Figure 1A (Figure 1B).

Next, we characterized the potential apoptotic effects of these two inhibitors using flow cytometry assay. As shown in Figure 1C, DU145, PC3 and ARCaP_E_ cells showed enhanced radio-sensitivity following increasing doses of AG1024 and/or Erlotinib, while ARCaP_M_ was only sensitive to AG1024, but not to Erlotinib. Combined treatment with Erlotinib and AG1024 synergistically radio-sensitize DU145, PC3 and ARCaP_E_ cells, but not ARCaP_M_ cells. When comparing DU145 cells to PC3 cells, we found that the former are more sensitive to Erlotinib than the latter, given that 10 µM Erlotinib induced a robust apoptotic response over 1 µM Erlotinib in DU145 cells, while not much increase in apoptosis was observed in PC3 cells by the same dosages of Erlotinib. Similar results were also achieved with siRNA targeting EGFR and/or IGF1R (Figure 1D).

### Enhancement of radio-sensitivity by EGFR and IGF1R inhibitors through impairment of DNA DSB repair

**Figure 2:**
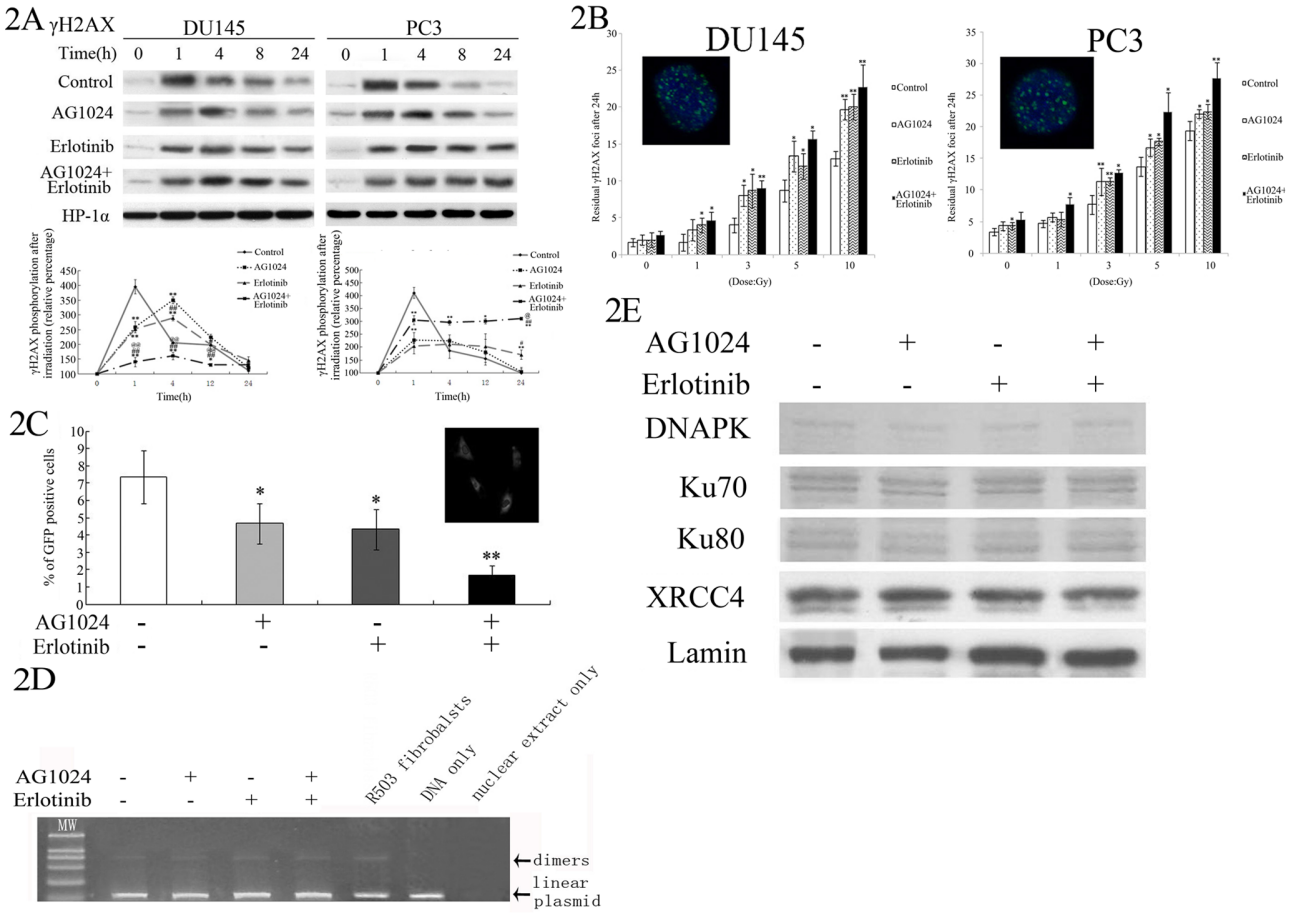
Effects of EGFR and IGF1R inhibitors on regulation of DSB repair capacity through suppression of HRR, but not NHEJ. A, DSB marker γH2AX expression time course after irradiation: Cells were serum starved overnight, then treated with 10 µM AG1024 and/or 10 µM Erlotinib. Western-blot of cells irradiated at the dosage of 2 Gy, and harvested at the indicated time points. HP-1α protein was used as a loading control. The data were then quantified by Image J software and expressed as percentage of the control. B, Immunofluorescence detection of γH2AX foci formation after irradiation. The cells were treated with or without AG1024 and/or Erlotinib for 1 h, and irradiated at different dosages. The cells were fixed after 24 h. The insets show representative images with green γH2AX signal and blue DAPI nuclear staining. C, DSB repair assay for HRR. Serum-starved DU145 cells in absence or presence of AG1024 (10 µM) or Erlotinib (10 µM) were transiently transfected with two vectors, one expressing I-SceI cDNA to generate DSBs of GFP cDNA, and another expressing red fluorescent protein with a mitochondrial localization signal to indicate transfection efficiency. DNA repair by HRR was evaluated by the ratio of cells with both red and green signals to those with only red signals. The insets show representative images of cells positive and negative for HRR. Data were presented as mean ± SD of the percentage of cells positive for DNA repair by HRR from three independent experiments. D, DSB repair assay for NHEJ. DU145 cells pre-treated with or without AG1024 (10 µM), Erlotinib (10 µM) or both. Corresponding nuclear extracts were incubated with the linearized plasmid pBluscript KS(+) in a cell-free system to measure NHEJ. The linearized plasmid DNA without treatment with the nuclear extracts (DNA only) and the nuclear extract from the control cells without the plasmid (nuclear extract only) were used the negative control. Nuclear extract from R503 fibroblasts was used as a positive control. Arrows indicate positions of linearized plasmid and dimers. E, Western-blot assay detecting NHEJ related protein. DU145 cells pre-treated with or without AG1024 (10 µM), Erlotinib (10 µM) or both, and then irradiated for NHEJ-related nuclear protein (DNAPK, K70/80, XRCC4). Lamin was used as a loading control. *P<0.05, **P<0.01, as compared to the control cells.

To understand the molecular mechanisms underlying enhanced radio-sensitivity of cancer cells in response to AG1024 and/or Erlotinib treatment, we determined the capacity of DNA double-strand break (DSB) in DU145 and PC3 cells, since DSB exerts the most lethal effect on cells induced by γ-irradiation. By monitoring the level of histone protein H2AX phosphorylation on the C-terminal serine 139 residue, also known as γH2AX, a well-known and sensitive DSB marker, we demonstrated that there was a rapid and robust phosphorylation of H2AX at 1 h after irradiation in both vehicle-treated control DU145 and PC3 cells (Figure 2A), which quickly declined at 4 h, and returned to the basal level at 24 h, implying the accomplishment of DSB repair. In contrast, tumor cells pre-treated with AG1024, Erlotinib, or both showed a delayed H2AX phosphorylation peak at 4 hour, and continuous H2AX phosphorylation till 24 h after irradiation, suggesting impairment of DSB repair after AG1024 and Erlotinib treatment (Figure 2A, B). Moreover, there was a significant difference between AG1024+Erlotinib group and AG1024 or Erlotinib group (5 and 10 Gy, P<0.01), indicating an additive effect of co-inhibtion on the DSB repair process (Figure 2B).

### Effects of EGFR and IGF1R inhibitors on impairment of DSB repair through inhibition of HRR, but not of NHEJ

To differentiate whether the impaired DSB repair was due to a defect in HRR or NHEJ, the two major pathways for DSB repair, we quantitatively assessed HRR using the pDR-GFP recombination reporter system and examined NHEJ using an *in vitro* cell-free assay [20]. As shown in Figure 2C, for DU145 cells, combined treatment with AG1024 and Erlotinib potently reduced HRR level when compared to the vehicle control or each agent alone (P<0.05). In contrast, the nuclear extract treated with individual inhibitor or both were unable to alter the *in vitro* ligation of pBluescript KS (+), when compared to the nuclear extract from vehicle control cells (Figure 2D). For this analysis, the nuclear extract from R503 cells was used as the positive control, as demonstrated by Yang et al [23]. In addition, expression of NHEJ-related DNA repair proteins, including DNA-PK, Ku70, Ku80 and XRCC4, were not affected by AG1024, Erlotinib or both in DU145 cells (Figure 2E), suggesting that inhibition of these two signaling pathways did not impair NHEJ-initiated DNA DSB repair, one of the most common forms of DSB repair in mammalian cells.

### Suppression of signal crosstalk at multiple levels by inhibiting both EGFR and IGF1R

**Figure 3:**
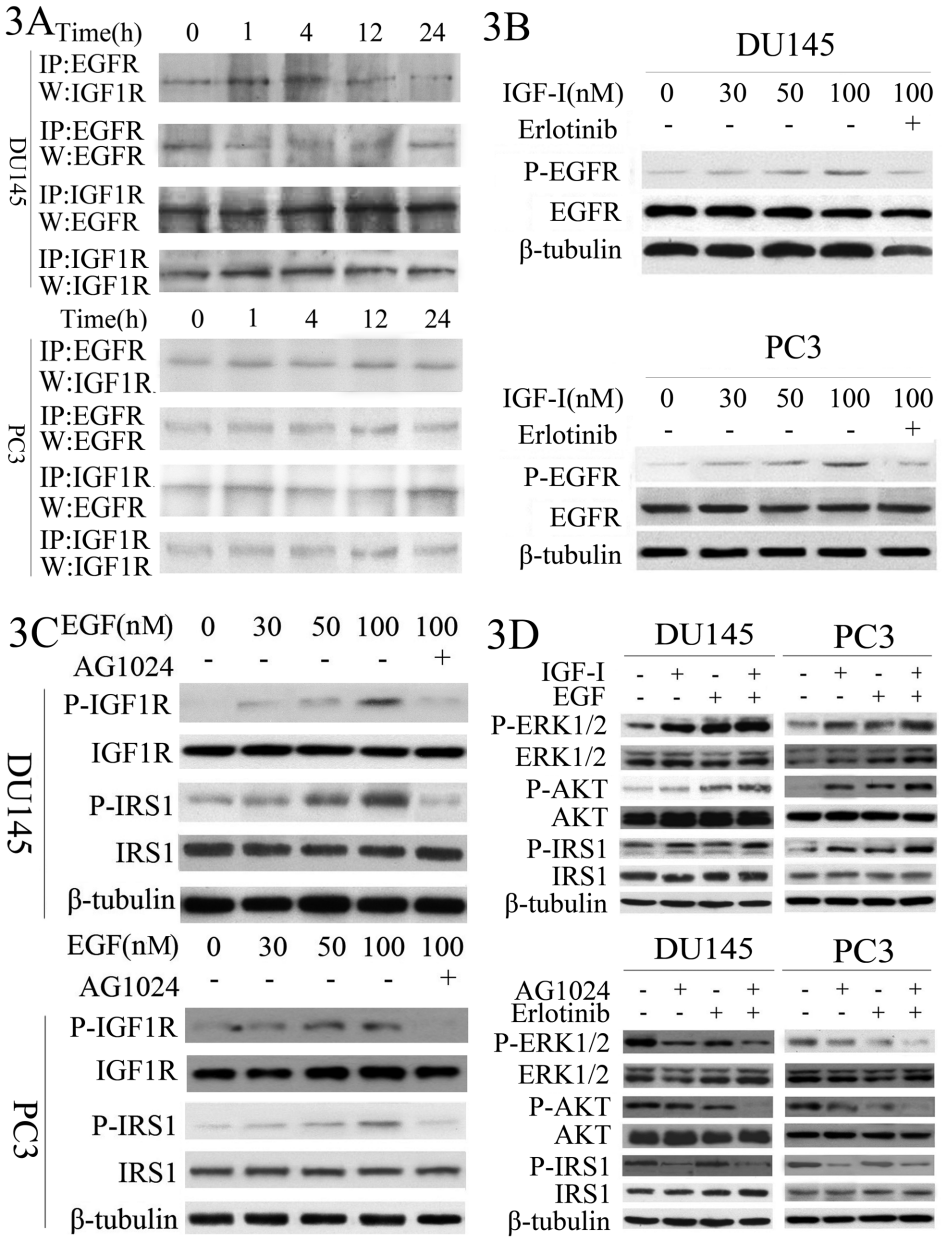
Effects of EGFR and IGF1R inhibitors on crosstalk between EGFR and IGF1R signaling pathways at multiple levels. **A**, Immunoprecipitation detecting EGFR/IGF1R interaction. DU145 and PC3 cells were irradiated at the dose of 2 Gy. Then cellular proteins were extracted, and subjected to immunoprecipitation assay detecting EGFR and IGF1R interaction. **B**, IGF-I activates EGFR in DU145 and PC3 cells. Cells were serum-starved overnight, and then subjected to IGF-I stimulation for 30 min. EGFR phosphorylation was detected by Western blot. **C**, EGF activates IRS1, the key factor of IGF1R signal pathway in DU145 and PC3 cells. Cells were serum-starved overnight, and then subjected to EGF stimulation for 30 min. IRS1 phosphorylation was detected by Western blot. **D**, MAPK or AKT activation after EGFR and IGF1R simulation of inhibition. DU145 and PC3 cells were treated with or without AG1024 (10 µM), Erlotinib (10 µM), AG1024 plus Erlotinib; IGF (50 ng/mL), EGF (50 ng/mL), or EGF plus IGF for 30 min. Then ERK1/2 expression and phosphrylation, AKT expression and phosphrylation, IRS1 expression and phosphrylation was determined by Western blotting. β-tubulin was used as a loading control.

Previous studies revealed extensive crosstalk between EGFR and IGF1R signaling pathway at multiple levels [14]. Our data above have demonstrated that co-inhibition of EGFR and IGF1R could additively radio-sensitize PC cells through the impairment of HRR DSB repair. To further investigate how the interaction between the two receptors affects DSB repair, we performed immunoprecipitation-Western-blot to examine the physical interaction between EGFR and IGF1R in DU145 and PC3 cells following γ-irradiation. Our data showed that without irradiation, these two receptors interacted with each other in both tumor cell lines and that this association was not significantly altered at 24 h after irradiation (Figure 3A). Moreover, we determined the downstream signal transduction of one receptor in response to the ligand of the other receptor. As shown in Figure 3B, for DU145 and PC3 cells, phosphorylation of EGFR was increased in a dose-dependent manner to IGF-I treatment in both cells but was significantly reduced upon co-treatment with the EGFR inhibitor, Erlotinib. Similarly, phosphorylation of IRS1, the major IGF1R signaling molecule, was enhanced after treatment with increasing concentrations of EGF, but was inhibited after addition of AG1024 (Figure 3C). In addition, we found that the ligand for each receptor, i.e., IGF-I and EGF, was sufficient to activate these target genes in DU145 and PC3 cells. However, the most robust activation was achieved with co-treatment with both IGF-I and EGF, whereas blockage of these receptors signaling with either AG1024 or Erlotinib was able to reduce activation of these target genes, but the most potent reduction was observed in DU145 and PC3 cells pre-treated with both inhibitors (Figure 3D). These data suggest that co-treatment with EGFR and IGF1R inhibitors was able to suppress their crosstalk and in turn block their downstream signaling, including PI3K/AKT pathway and MAPK pathway.

### Effects of EGFR and IGF1R inhibitors on suppression of IRS1/Rad51-mediated homologous recombination

**Figure 4:**
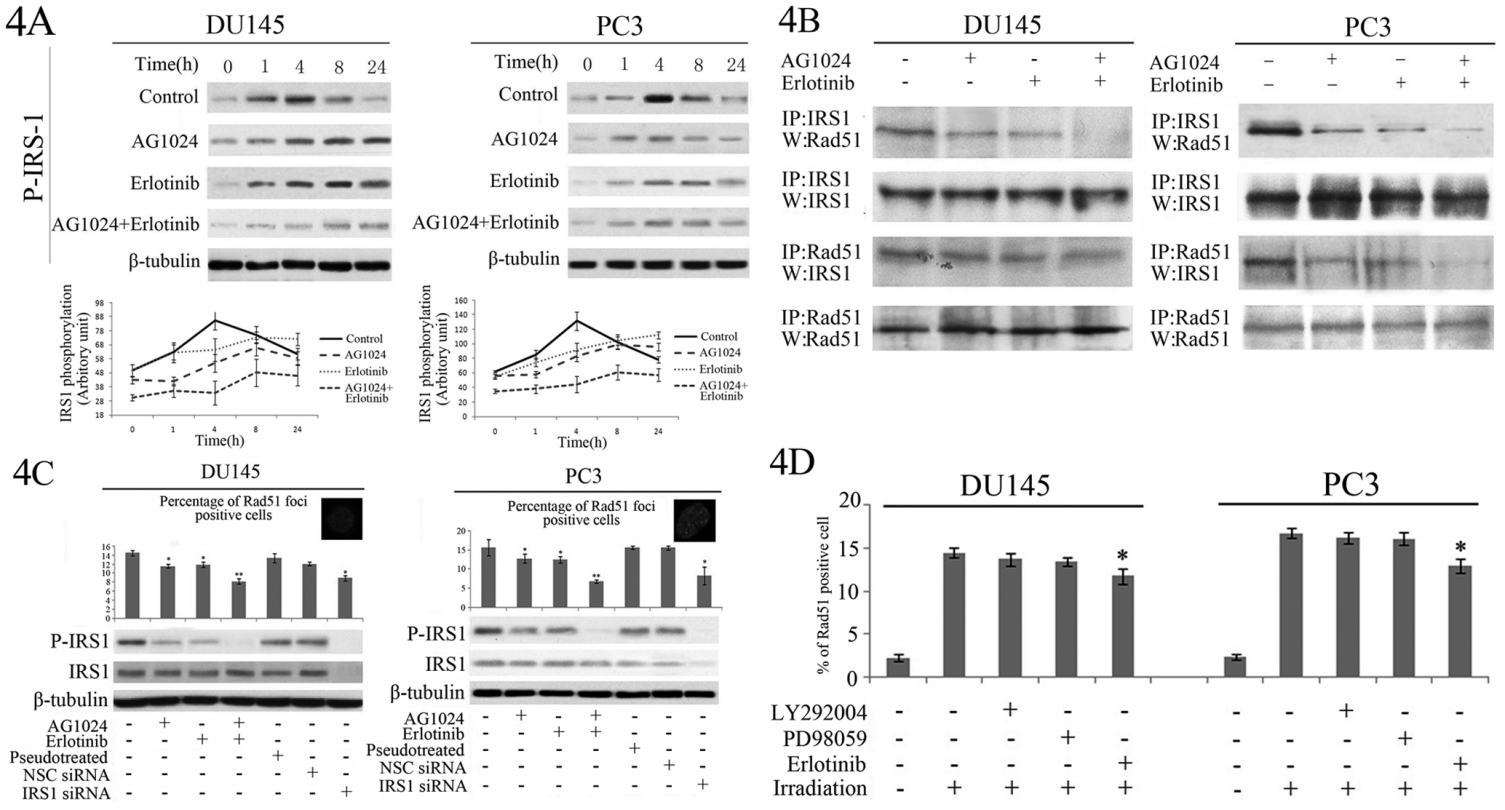
Effects of EGFR and IGF1R inhibitors in regulation of IRS1/Rad51-mediated HRR. **A**, Western blot showing level of p-IRS1 at different time points after 2 Gy γ-irradiation of DU145 and PC3 cells pre-treated with or without AG1024 (10 µM), Erlotinib (10 µM), or both for 24 h. β-tubulin was used as a loading control. The representative gel image is shown at the top and quantification p-IRS1/β-tubulin ratio from three independent experiments shown in the bottom. **B**, Immunoprecipitation showing interaction between IRS1 and Rad51 in the nuclear extract of DU145 and PC3 cells. **C**, The formation of nuclear Rad51 foci as analyzed by immunofluorescence staining. DU145 and PC3 cells were maintained with or without AG1024 (10 µM), Erlotinib (10 µM) or both (left four bars), mock-transfected (pseudotreated), or transfected with non-specific control siRNA (NSC siRNA) or with IRS1 siRNA (right three bars). The insets show representative images with green Rad51 signal and blue DAPI nuclear staining. The data were presented as mean ± SD from three independent experiments. *P<0.05, as compared to AG1024/Erlotinib- treated or pseudotreated cells; **P<0.01, as compared to AG1024- or Erlotinib-treated cells. **D**, Rad51 immunofluorescence staining of DU145 and PC3 cells treated with LY292004, PD98059, or Erlotinib followed by γ-irradiation. The nuclear Rad51 foci formation was determined in the same as in C. Insets show representative images with green Rad51 signal and blue DAPI nuclear staining. Data were presented as mean ± SD from three independent experiments. *P<0.05, as compared to the irradiated-only cells.

Our current data on AG1024 and Erlotinib regulating IRS1 phosphorylation/activation implicated the potential involvement of IRS1/Rad51-mediated HRR in response to γ irradiation in PC cells, as previous study also indicated [24]. Indeed, our data showed that IRS1 phosphorylation was induced by γ-irradiation with peak activation achieved at 4 h after γ-irradiation in DU145 and PC3 cells, while the peak of IRS1 phosphorylation was delayed to 8 h after γ-irradiation following treatment with AG1024 or Erlotinib alone. In contrast, co-treatment with both AG1024 and Erlotinib significantly reduced IRS1 activation not only at the basal level (0 h post-irradiation) but also at all time points up to 24 h after irradiation (Figure 4A).

Next, we determined the physical interaction between IRS1 and Rad51 in the nuclear fraction of cells followed γ-irradiation using immunoprecipitation. As shown in Figure 4B, treatment with vehicle, AG1024, Erlotinib or AG1024 plus Erlotinib did not significantly change levels of IRS1 or Rad51 protein, whereas levels of IRS1-associated with Rad51 were dramatically reduced in cells treated with AG1024 plus Erlotinib, which is consistent with the significantly reduced level of phospho-IRS1 in these cells. These data demonstrated that AG1024 plus Erlotinib treatment suppressed IRS1 interaction with Rad51, indicating EGFR and IGF1R co-inhibition could inhibit DNA double strand break repair by inhibition of HRR.

In addition, we also determined the subcellular localization of Rad51 that forms nuclear foci at sites of DNA lesions. Our data showed that approximately 15% of vehicle-treated control cells were positive for Rad51 nuclear foci after exposure to γ-irradiation. The percentage of cells with positive foci was significantly reduced when the cells were pre-treated with either AG1024 or Erlotinib (P<0.05), and was more robustly reduced in those co-treated with AG1024 plus Erlotinib cells (P<0.01, Figure 4C). The positivity of Rad51 nuclear foci was associated with the levels of IRS1 phosphorylation. The positive relationship between IRS1 activation and positivity of Rad51 nuclear foci was further supported by targeting IRS1 using IRS1 siRNA. When compared to mock-transfected cells or those transfected with non-silencing control siRNA (NSC siRNA), IRS1 siRNA significantly reduced the total IRS1, IRS1 phosphorylation, and the number of positive cells with Rad51 nuclei foci (P<0.05, Figure 4C). To further characterize the regulation of Rad51 nuclear foci formation, we pre-treated cancer cells with EGFR inhibitor Erlotinib, PI3K inhibitor LY292004, or ERK inhibitor PD98095 and found that only pre-treatment with Erlotinib led to significant reduction of Rad51-positive nuclear foci (P<0.05, Figure 4D), implying that regulation of Rad51-mediated HRR depends on upstream signaling molecule(s) such as IRS1, rather than downstream ones, such as PI3K/AKT or ERK.

### Effects of EGFR and IGF1R inhibitors on sensitization of cancer cells to radiotherapy *in vivo*

**Figure 5:**
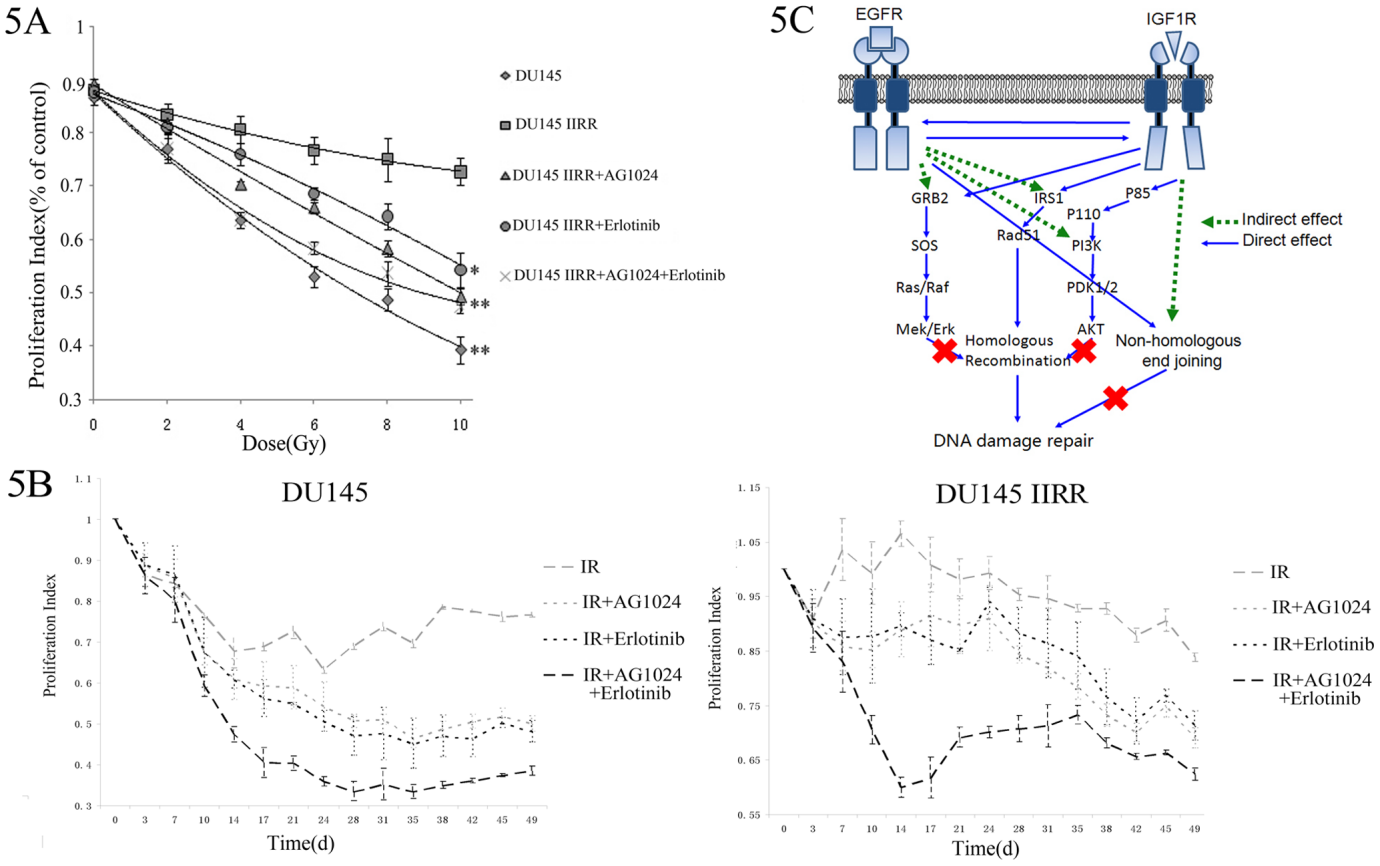
*In vivo* effects of EGFR and IGF1R inhibitors in sensitivity of prostate cancer to radiotherapy. **A**, Dose-proliferation relation of nude mouse xenograft growth. Prostate cancer DU145 and irradiation-resistant DU145IIRR cells were subcutaneously injected into nude mice to form xenograft tumors and followed by indicated treatments and γ-irradiation. At day 40 after treatment, the tumor growth in different mice was presented as proliferation index. The data were presented as mean ± SD from three independent experiments. *P<0.05, **P<0.01, as compared to the control cells. **B**, Time-proliferation relation of nude mouse xenograft growth. At different time points after treatment, the tumor growth in different groups was presented as the proliferation index. **C**, Schematic diagram illustrating co-inhibition of EGFR and IGF1R sensitizes prostate cancer to radiotherapy via inhibition of HRR DNA damage repair. EGFR and IGF1R are able to directly interact with each other to activate multiple downstream genes, such as IRS1, ERK, and AKT. Upon γ-irradiation, this interaction was not affected, while facilitating NHEJ and IRS1/Rad51-mediated HRR for DNA double-strand break repair. Simultaneously blockage of EGFR and IGF1R gene pathways disrupts their crosstalk on multiple levels; for example, suppression of EGFR/IGF1R interaction, attenuation of IRS1 phosphorylation and impairment of IRS1/Rad51-mediated HRR. However, NHEJ was not affected by EGFR and IGF1R inhibition.

Our results so far demonstrated the effects of these EGFR and IGF1R inhibitors on PC cells with or without γ-irradiation *in vitro*. Next, we further determined their *in vivo* effects using the nude mouse xenografts model. We injected parental DU145 cells as well as irradiation-resistant sublines DU145-IIRR into nude mice. As shown in Figure 5A, tumors derived from DU145-IIRR were significantly more resistant to γ-irradiation than those derived from the parental DU145. However, this resistance was diminished after treatment with either Erlotinib or AG1024, or both in a time-dependent manner. As shown in the left panel of Figure 5B, γ-irradiation significantly reduced *in vivo* tumor growth of parental DU145 (P<0.05, as compared to the control mice), which was further reduced by treatment with AG1024 or Erlotinib (P<0.05, as compared to γ-irradiation mice), and by co-treatment with AG1024 plus Erlotinib (P<0.05, as compared to γ-irradiation plus AG1024 or γ-irradiation plus Erlotinib mice). Although tumor growth was not dramatically affected by irradiation over time with DU145-IIRS cell injections, treatment with AG1024, Erlotinib, or both sensitized the tumors to γ-irradiation. Combined treatment with both inhibitors significantly inhibited tumor growth, comparing with any one of these inhibitor treatment, indicating the synergistic growth inhibitive effect.

## Discussion

In this study, we investigated whether co-targeting of EGFR and IGF1R could sensitize PC cells to γ-irradiation. Our current data provide evidence that crosstalk between EGFR and IGF1R occurs not only at the cell surface via the receptor interaction, but also through their crosstalk, and thus targeting both pathways interferes HRR of DSB repair by modulating IRS1 and Rad51 interaction, resulting in radio-sensitization of PC cells.

Radiotherapy induces multiple types of damage in genomic DNA, including single-strand breaks (SSB), DSB, base alterations, DNA-DNA, and DNA-protein crosslinks. Without proper DNA repair capacity, cells undergo apoptosis, mitotic catastrophe, autophagy, cellular senescence, or even carcinogensis [25]. However, in tumor cells after radiation therapy, enhanced DNA repair capacity such as HRR or NHEJ leads to resistance to radiotherapy. HRR is a template-driven and thus error-free DNA repair mechanism that involves many proteins including Rad51, p53, BRCA2, BLM, and RPA. NHEJ is a more error-prone mechanism that operates with DNA repair proteins such as Ku70/80, XRCC4, XRCC1, DNA-PK, and XLF [25]. In the current study, we showed that inhibition of both EGFR and IGF1R was able to suppress HRR repair of damaged DNA after γ-irradiation, but not NHEJ repair. This explains why the combination of these two inhibitors could synergistically inhibit HRR, but only showing additive effect on DSB repair inhibition, consistent with previous studies that NHEJ is the main mechanism of DSB repair [26].

Indeed, studies over the past decade have suggested several mechanisms of regulation between radiation-induced EGFR and IGF1R signaling and DSB repair. Both signaling pathways including the downstream signaling cascades are activated in response to ionizing radiation [27]. Upon their activation, the PI3K-AKT and Ras-MAPK pathways present specific activity toward resistance to chemo- and radio-therapy, in addition to their regulatory capability on cell viability, apoptosis and cell proliferation [28], [29]: The Ras-MAPK pathway stimulates production of EGFR ligand and in turn activates EGFR in an autocrine manner, while PI3K-AKT pathway directly activates the catalytic subunit of DNA-PK, the essential component for NHEJ machinery. The nuclei-localized EGFR protein following activation will facilitate nuclear localization of DNA-PK and therefore, promote NHEJ in addition to transcriptional regulation of XRCC1 and BER expression. These studies suggest a preferential link between EGFR signaling and NHEJ-mediated DNA repair. However, in IGF1R pathway, Trojanek *et al.* demonstrated that upon IGF-I stimulation, IRS1, the major IGF1R substrate, binds with Rad51, stimulates nuclear localization of the latter to the site of damaged DNA, and promotes HRR [24].

Altogether, both EGFR and IGF1R are important in repairing irradiation-damaged DNA. Indeed, our current study found that in multiple epithelial PC cells, simultaneous blockage of EGFR and IGFR dramatically reduced cell viability by induction of apoptosis. We also noticed differential sensitivities of DU145 and PC3 to IGF1R and EGFR inhibitors, which may result from the different PTEN status in these two cell lines, since DU145 is positive for while PC3, negative for PTEN.

Furthermore, in this study, we demonstrated mechanisms of crosstalk between EGFR and IGF1R signaling pathways at multiple levels in PC cells. We showed that these two receptors physically interact, and, the activation of one receptor led to stimulation of the other. Moreover, several downstream targets, including IRS1, AKT and ERK were synergistically activated by co-stimulation of both receptors (Fig. 3D). These data revealed a novel strategy by using inhibitors of both receptors to effectively treat PC and sensitize cells to γ-irradiation therapy. However, further studies will be required before this can be translated into clinical practice. Up to now, the main method of to treat PC is hormone therapy. Prostate cancers are mostly androgen-sensitive in the beginning, and inevitably transform to androgen-insensitive and become androgen-refractory as PC progresses. Therefore, PC patients differ in androgen sensitivity. In this research, we used androgen-independent PC cell lines in this research. Whether our findings could be extended to androgen-sensitive or androgen-refractory PC needs to be further investigated.

In summary, we provide the first experimental evidence that in androgen-independent PC cells, crosstalk between EGFR and IGF1R contributes to radiation-induced DSB repair through the suppression of HRR via IRS1/Rad51 signal pathway. Even though other DNA repair mechanisms, such as those for DNA-SSB, altered DNA bases, and DNA-DNA or DNA-protein crosslink, may function following radiation-induced DNA lesions, targeting both EGFR and IGF1R activities may provide a novel approach for improving the efficacy of anti-PC radiotherapy.

## Supporting Information

Figure S1 **Effects of EGFR and IGF1R inhibitors in suppression of EGFR and IGF1R phosphorylation in DU145 and PC3 cells.** Western blot analysis was performed to determine phosphorylated IGF1R and EGFR levels in prostate cancer cells DU145 and PC3, pre-treated with or without AG1024 (10 µM), Erlotinib (10 µM) or both for 1 h.
(TIF)

Figure S2 **Effects of IGF1R and EGFR siRNA in knockdown of IGF1R and EGFR in DU145 and PC3 cells.** None-silencing control (NSC) siRNA, IGF1R siRNA and EGFR siRNA were transfected into DU145 and PC3 cells by Oligafectamine method. Total cell proteins were extracted at 24 h after transfection. Western blot analysis was used to detect IGF1R and EGFR expression in DU145 and PC3.
(TIF)
