# Supplementary figures and images for: Inhibition of Both EGFR and IGF1R Sensitized Prostate Cancer Cells to Radiation by Synergistic Suppression of DNA Homologous Recombination Repair
Source: PLoS One. 2013 Aug 12;8(8):e68784. doi: 10.1371/journal.pone.0068784 (PMC3741308; doi:10.1371/journal.pone.0068784)

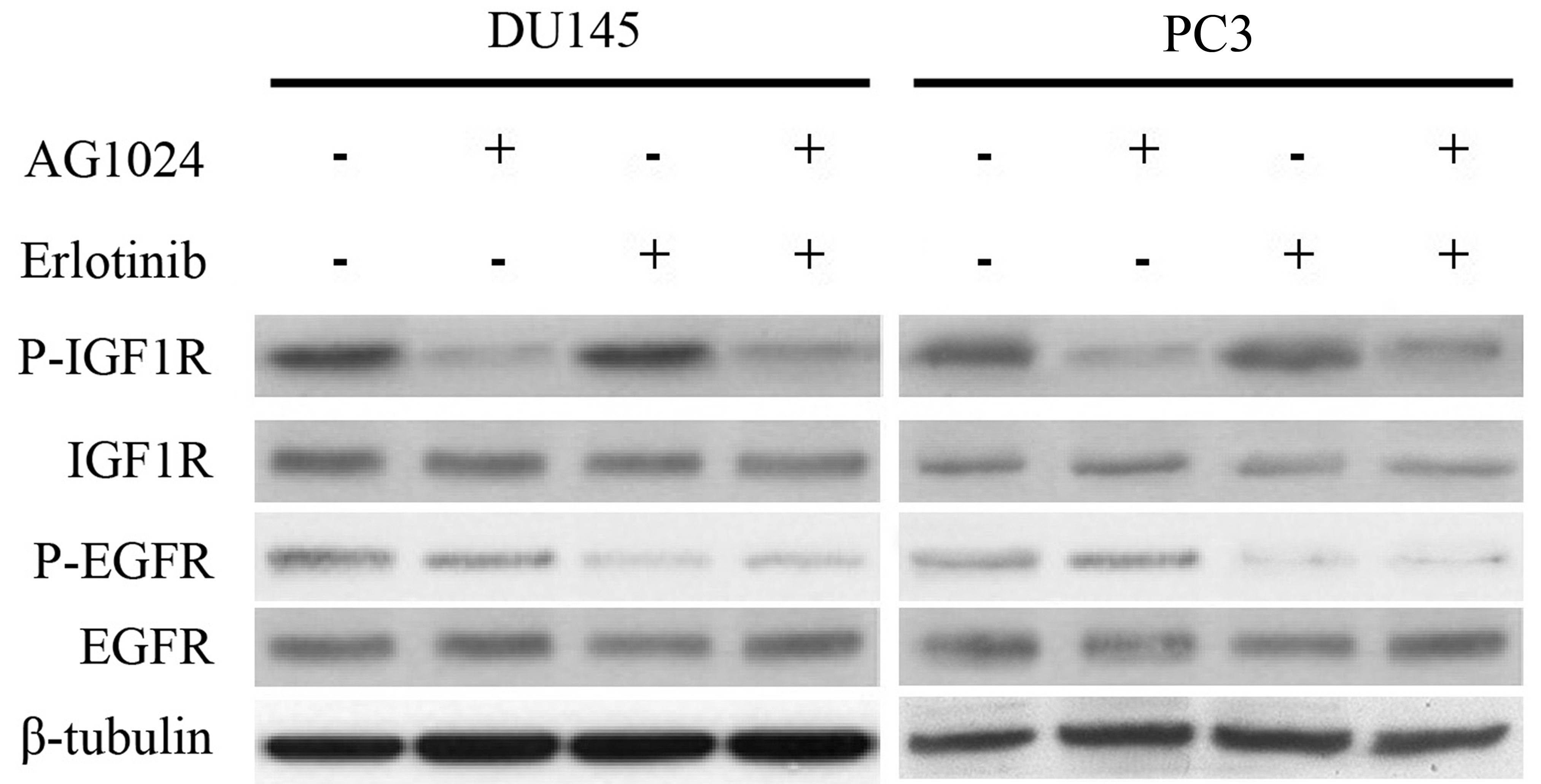

Supplement: Figure S1 — Effects of EGFR and IGF1R inhibitors in suppression of EGFR and IGF1R phosphorylation in DU145 and PC3 cells. Western blot analysis was performed to determine phosphorylated IGF1R and EGFR levels in prostate cancer cells DU145 and PC3, pre-treated with or without AG1024 (10 µM), Erlotinib (10 µM) or both for 1 h. (TIF) [file pone.0068784.s001.tif]

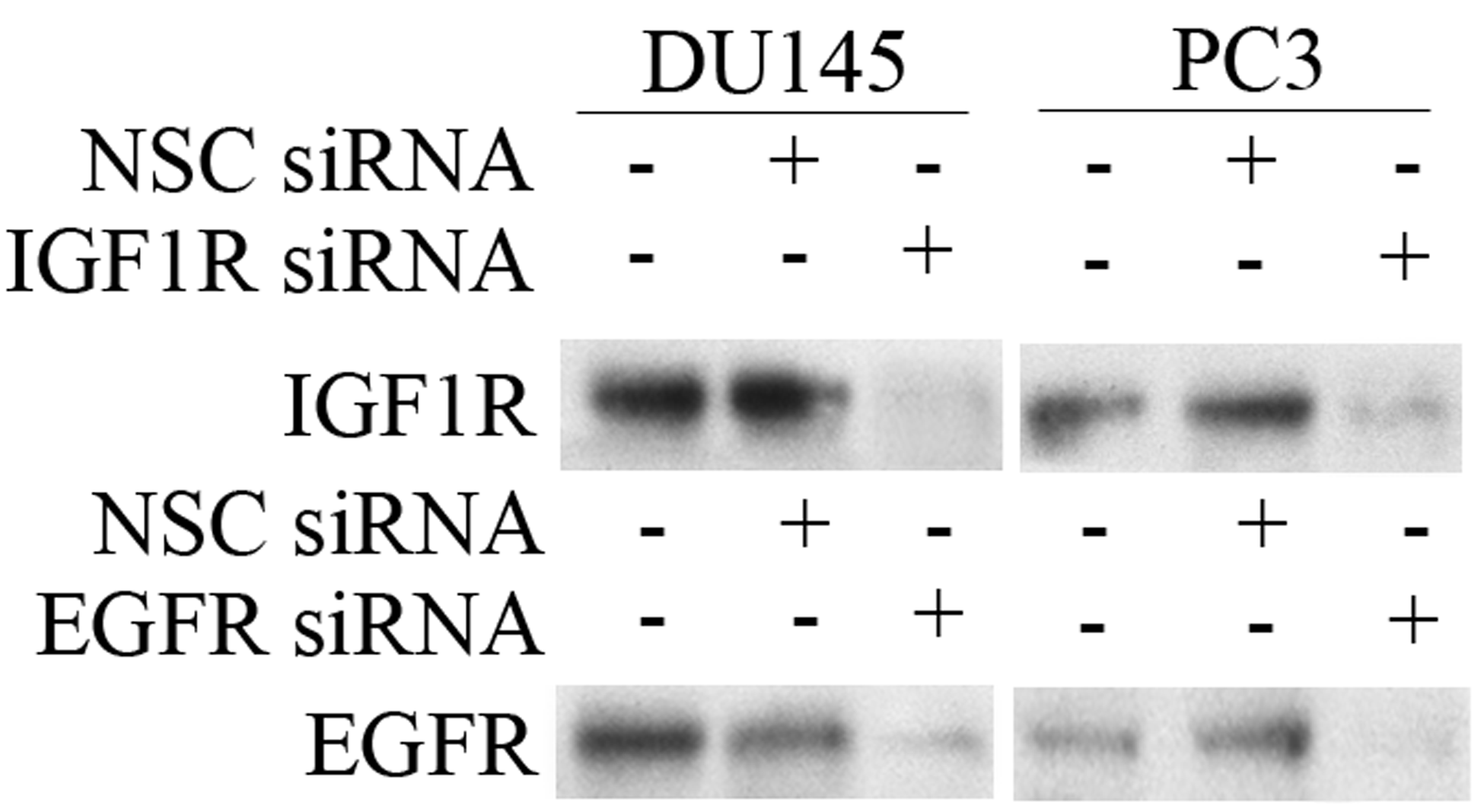

Supplement: Figure S2 — Effects of IGF1R and EGFR siRNA in knockdown of IGF1R and EGFR in DU145 and PC3 cells. None-silencing control (NSC) siRNA, IGF1R siRNA and EGFR siRNA were transfected into DU145 and PC3 cells by Oligafectamine method. Total cell proteins were extracted at 24 h after transfection. Western blot analysis was used to detect IGF1R and EGFR expression in DU145 and PC3. (TIF) [file pone.0068784.s002.tif]
